# Supplementary material for: Conditional chemoconnectomics (cCCTomics) as a strategy for efficient and conditional targeting of chemical transmission
Source: eLife. 2024 Apr 30;12:RP91927. doi: 10.7554/eLife.91927 (PMC11060718; doi:10.7554/eLife.91927)
Supplement: Figure 3—source data 3. [file elife-91927-fig3-data3.zip › Figure 3 source data3 part 2.zip!/Figure 3-Source data 3-part2/1025-13-CRISPResso_Report_ack040l3.zip!/CRISPResso_on_1025_14/4a.Combined_insertion_deletion_substitution_locations.pdf]

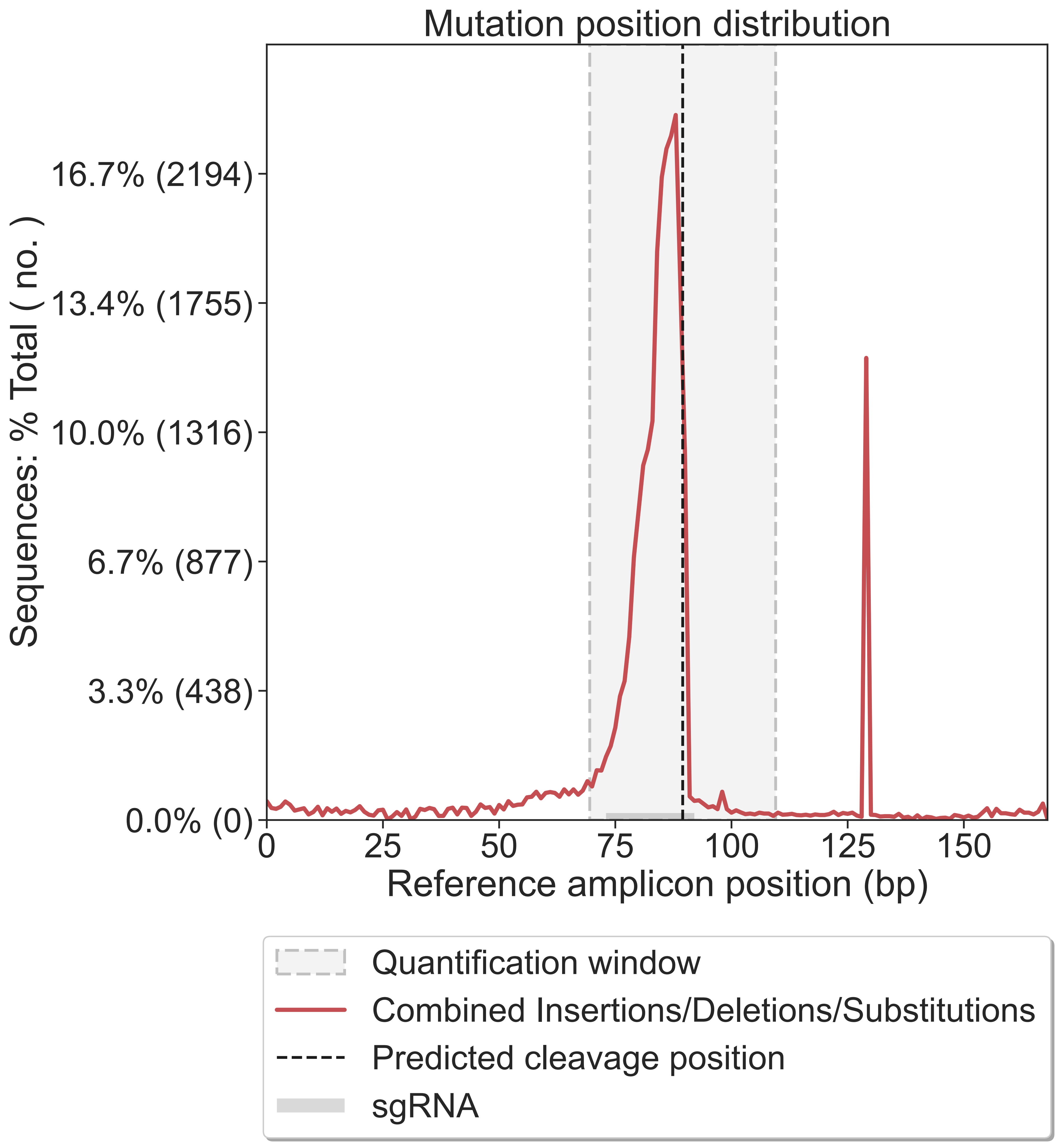

Mutation position distribution
Sequences: % Total (no.)
16.7% (2194)
13.4% (1755)
10.0% (1316)
6.7% (877)
3.3% (438)
0.0% (0)
0
25
50
75
100
125
150
Reference amplicon position (bp)
Quantification window
Combined Insertions/Deletions/Substitutions
Predicted cleavage position
sgRNA
